# Errata in No. 1

**Published:** 1838-01-17

**Authors:** 


ERRATA IN No. 1.

In Dr. Pepper's case, p. 6, line 11 from bottom, for *no alteration*, read *no ulceration*.

In the domestic Summary, p. 15, art., Hysterotomy, line 21 from top, for *inferior strait* read *superior strait*.

In the Foreign Summary, p. 16, line 33 from top, for *ophineter*, read *sphincter*.

